# Understanding heart failure; explaining telehealth – a hermeneutic systematic review

**DOI:** 10.1186/s12872-017-0594-2

**Published:** 2017-06-14

**Authors:** Trisha Greenhalgh, Christine A’Court, Sara Shaw

**Affiliations:** Nuffield Department of Primary Care Health Sciences, University of Oxford, Radcliffe Primary Care Building, Woodstock Rd, Oxford, OX2 6GG UK

**Keywords:** Heart failure, Telehealth, Systematic review, Hermeneutic review, Patient experience

## Abstract

**Background:**

Enthusiasts for telehealth extol its potential for supporting heart failure management. But randomised trials have been slow to recruit and produced conflicting findings; real-world roll-out has been slow. We sought to inform policy by making sense of a complex literature on heart failure and its remote management.

**Methods:**

Through database searching and citation tracking, we identified 7 systematic reviews of systematic reviews, 32 systematic reviews (including 17 meta-analyses and 8 qualitative reviews); six mega-trials and over 60 additional relevant empirical studies and commentaries. We synthesised these using Boell’s hermeneutic methodology for systematic review, which emphasises the quest for understanding.

**Results:**

Heart failure is a complex and serious condition with frequent co-morbidity and diverse manifestations including severe tiredness. Patients are often frightened, bewildered, socially isolated and variably able to self-manage. Remote monitoring technologies are many and varied; they create new forms of knowledge and new possibilities for care but require fundamental changes to clinical roles and service models and place substantial burdens on patients, carers and staff. The policy innovation of remote biomarker monitoring enabling timely adjustment of medication, mediated by “activated” patients, is based on a modernist vision of efficient, rational, technology-mediated and guideline-driven (“cold”) care. It contrasts with relationship-based (“warm”) care valued by some clinicians and by patients who are older, sicker and less technically savvy. Limited uptake of telehealth can be analysed in terms of key tensions: between tidy, “textbook” heart failure and the reality of multiple comorbidities; between basic and intensive telehealth; between activated, well-supported patients and vulnerable, unsupported ones; between “cold” and “warm” telehealth; and between fixed and agile care programmes.

**Conclusion:**

The limited adoption of telehealth for heart failure has complex clinical, professional and institutional causes, which are unlikely to be elucidated by adding more randomised trials of technology-on versus technology-off to an already-crowded literature. An alternative approach is proposed, based on naturalistic study designs, application of social and organisational theory, and co-design of new service models based on socio-technical principles. Conventional systematic reviews (whose goal is synthesising data) can be usefully supplemented by hermeneutic reviews (whose goal is deepening understanding).

**Electronic supplementary material:**

The online version of this article (doi:10.1186/s12872-017-0594-2) contains supplementary material, which is available to authorized users.

## Background

The use of telehealth technology in heart failure management remains controversial, with some clinicians and policymakers strongly enthusiastic [1–4] and others unconvinced or opposed [5–7]. Despite dozens of randomised controlled trials (including several megatrials [8–13]), over 20 systematic reviews and meta-analyses of clinical trials [14–33] and (at last count) 7 systematic reviews of systematic reviews [6, 34–39], as well as studies of cost-effectiveness [21, 40–43], the patient experience [18, 24, 44–47] and service organisation [32, 48–51], there is limited agreement on which outcomes matter; the extent to which telehealth adds benefit over usual care; how best (if at all) to change service models to support telehealth; and whether and in what circumstances telehealth is cost-effective.

In the context of high and rising prevalence of heart failure [52–54] and its unenviable position atop the league table of reasons for hospital readmission [55], recruitment of heart failure patients to clinical trials of telehealth is consistently poor [17, 32, 56–58]; roll-out and scale-up of telehealth services for heart failure in real-world settings is slow [32, 49, 59–63]; and robust business models are lacking [5]. Systematic reviews and meta-analyses have been criticised for major methodological flaws [6, 33, 36, 38, 39, 64, 65] and economic evaluations for their questionable and/or unstated assumptions [42]. The service and regulatory context is under-researched.

In sum, the literature on telehealth in heart failure is a policymaker’s nightmare: vast, fragmented, heterogeneous, of variable quality and with no clear answers to the question of what technologies, supported by what service infrastructure, to provide for whom.

In a paper entitled ‘What makes an academic paper useful for health policy?’, Chris Whitty emphasised that the most useful contribution academics can make to a contested topic area is a succinct and integrative overview incorporating quantitative, qualitative and economic evidence which, above all, *makes sense* of the field [66]. In this study, we sought to produce a scholarly synthesis of the key questions, theoretical perspectives and empirical findings on the topic of telehealth in heart failure with a view to informing a new empirical study by our own team.

We began with five research questions, which we modified as the study progressed:

1. What is heart failure and what do we know about its prevalence, diagnosis, prognosis, co-morbidities and management?
2. What technologies are available to support remote clinical care in heart failure?
3. How might these technologies improve – or indeed worsen – the organisation, delivery and cost of heart failure services?
4. How might telehealth technologies and services influence, and be influenced by, the patient’s experience of heart failure and his or her capacity to cope with it?
5. What explains the low uptake of telehealth by patients, staff and organisations – both within and beyond the clinical trial setting?

### Study design and setting

The study had two phases: secondary research (hermeneutic systematic review, reported here) and a qualitative case study of telehealth, linked to a randomised controlled trial (to be reported elsewhere). These phases were overlapping; emerging findings from the review fed into the design of the qualitative study and influenced the data analysis.

## Method

The study was part of the SCALS (Studies in Co-creating Assisted Living Solutions) Senior Investigator Award to TG from the Wellcome Trust and was also supported by the knowledge translation component of the Oxford Biomedical Research Centre. Governance included 6-monthly meetings of an external steering group with an independent lay chair. Research ethics approval was not needed for the study reported here.

**Fig. 1:**
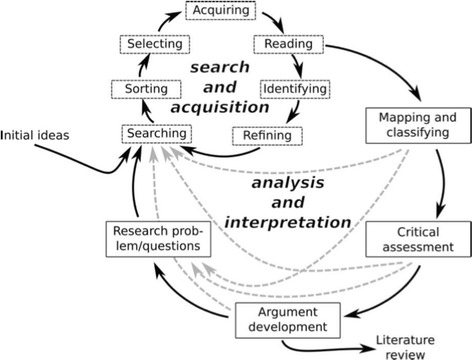
Hermeneutic systematic review. *Reproduced with permission from* [67]

We used an interpretive approach so as to meaningfully synthesise and critique the extensive existing literature. In their introduction to hermeneutic systematic review (shown diagrammatically in Fig. 1), Boell and Cecez-Kecmanovic observed that *“highly structured approaches [to systematic review] downplay the importance of reading and dialogical interaction between the literature and the researcher; continuing interpretation and questioning; critical assessment and imagination; argument development and writing – all highly intellectual and creative activities, seeking originality rather than replicability”* [67].

Hermeneutic review consists of two interlinked cycles: [a] accessing and interpreting the literature and [b] developing an argument. Searching is systematic but flexible and iterative. As sources accumulate, it becomes necessary to interpret, clarify and understand the emerging ideas and perspectives and to reject less relevant sources through progressive focusing [67]. This feeds into the lower cycle in Fig. 1.

TG began with a generic PubMed search for ‘heart failure’ and ‘telehealth’, including synonyms for both. She quickly identified a sample of highly-cited review articles covering both quantitative and qualitative research [5, 7, 17, 21, 38, 52, 65, 68–70]. She used citation tracking in Google Scholar to identify subsequent articles that had cited these seminal sources [71]. Of hundreds of potentially relevant titles and abstracts, she used one over-riding question to select a sample of papers for full-text analysis: is this paper likely to *add meaning* to our emerging overview of the field? To prevent the dataset becoming unmanageably large, systematic reviews (and critiques of these reviews) were used as far as possible. Selected individual papers were included to add richness to the synthesis.

CAC (a general practitioner with a special interest in cardiology) selected additional sources describing the current understanding of heart failure, with reference to national and European guidelines, selected conference abstracts and recently published reviews known to her. Peer reviewers selected by the editor pointed us to additional sources.

We stored and managed included studies on an Endnote database. We created a simple data extraction form to summarise key data, arguments and explanations from each source. Beginning with seminal reviews, we began to craft a narrative synthesis of the key questions, theories, methods, findings and scholarly arguments relevant to our research questions. We progressively refined this synthesis as further papers were added.

## Results

Included sources are summarised in Table 1 (the hermeneutic design precluded a conventional study flowchart). Findings fell into seven broad headings: heart failure as a clinical condition; heart failure as a lived experience; studies of self-management; descriptions of technologies; experimental trials of telehealth technologies; economic analyses; and implementation studies. We consider these in turn.

**Table 1:** Summary of included studies in the narrative review

General background	One textbook [53] plus background sections from other included papers
Heart failure as a clinical condition	Above plus 9 narrative reviews [5, 7, 33, 52, 55, 65, 73, 76, 83], 4 cohort studies [72, 82, 84–86], 1 national audit [80], guidance from professional societies [74, 75, 81, 117–119]
Heart failure as a lived experience	8 systematic reviews of qualitative or mixed-method studies [45, 90–95, 97], 5 additional qualitative studies [98–102], one theoretical paper [96], 1 commentary [104]
Self-management of heart failure	4 systematic reviews [18, 24, 44, 45], 2 realist reviews [46, 47], 2 additional qualitative studies [105, 106]
Technologies for remote monitoring of heart failure	3 narrative reviews [64, 65, 68], plus background sections from other included papers
Trials of telehealth in heart failure	7 meta-reviews (systematic reviews of systematic reviews) [6, 34–39], 20 systematic reviews of trials (16 with meta-analysis [14–29] and 4 without [30–33]), 6 M-trials [8–13], plus additional narrative reviews and commentaries, especially [33, 64, 65, 68, 70, 120, 121]
Economic analyses	5 systematic reviews of economic evaluations [21, 40–43] of which one included meta-analysis [41]
Implementation studies of telehealth in heart failure	3 systematic reviews [32, 48, 49], 2 narrative reviews [50, 51], 7 qualitative case studies (in 11 publications) of real-world implementation, including staff and patient experience [58, 60, 61, 87, 89, 101, 110, 111, 113, 114, 122], 3 national/international audits [62, 63, 123], 1 co-design study prior to RCT [112], 1 action research study of real-world implementation [60, 114], plus discussion sections from other included papers especially [5, 7]

### Heart failure as a clinical condition

Heart failure affects 1–4% of the adult population; it is commoner in ethnic minorities [5, 7, 52, 72]. It has multiple causes and complex pathophysiology (see Additional file 1). On average, four to five comorbidities add to symptom and treatment burden and influence prognosis, and co-existing frailty is common [73].

Early clinical trials focused on people with heart failure with reduced ejection fraction (HFREF) [52]. Increasingly, heart failure is defined primarily by its clinical characteristics even in research studies; recent telehealth trials have included participants who have heart failure with preserved ejection fraction (HFPEF). These issues are discussed in more detail in Additional file 1 [52].

The *incidence* (new cases per year) of heart failure in high-income countries has reportedly been stable for a generation, attributed to the falling incidence of coronary heart disease offset by improved survival from myocardial infarction (the commonest precursor to HFREF). However, its *prevalence* (total number of cases in a population) is rising because of improved disease-specific survival and general population ageing [7, 52, 53, 74, 75]. Furthermore, since HFPEF is largely a disease of the elderly that is strongly linked to hypertension, diabetes, obesity and atrial fibrillation, all of which are becoming commoner [76], some authors predict an impending increase in both incidence and prevalence of heart failure with a rising proportion of cases being HFPEF [77]. HFREF may also be set to increase in incidence as cancer patients who have received cardiotoxic chemotherapy survive long term [52].

Heart failure was traditionally managed with diuretics, digoxin and fluid restriction. Based on findings from numerous clinical trials, guidelines now recommend that all HFREF patients are prescribed the maximum tolerated dose of a beta-blocker and a drug acting on the renin-angiotensin system, with other drugs added according to severity [69, 78]. Efforts to achieve this are known as ‘up-titration’ – a clinically tricky task, since adverse effects are common with higher drug doses and with combination therapy [69]; polypharmacy is a serious risk [79]. The proportion of UK patients actually receiving the medication recommended by NICE guidelines for HFREF varied from 33% to 75% in one recent study, suggesting that there may be room for improvement [80]. A community-based study found a similar evidence-practice gap [72].

Management of heart failure with preserved ejection fraction (HFPEF) is controversial. Most trials (and most meta-analyses) to date have excluded such patients; trials that did include them are hard to interpret because of heterogeneous inclusion criteria, high withdrawal rates, small effect sizes and other limitations [69]. Diuretics, beta-blockers and drugs acting on the renin-angiotensin system may all improve aspects of morbidity but we could find no evidence that they affect mortality in HFPEF [69]. The receommended management strategy for HFPEF is twofold: careful reduction of fluid overload using diuretics and fluid restriction, which often entails a fine balancing act as the stiffened ventricle makes these patients vulnerable to over-diuresis, causing a critical reduction in cardiac output; and controlling predisposing conditions such as hypertension or diabetes [53, 78].

Implantable defibrillators, which do not affect underlying ventricular function, and atrioventricular synchronised biventricular pacemakers (also referred to as cardiac resynchronisation therapy), which do, have both been shown to improve survival in people with moderate to severe chronic heart failure, though the benefit-harm balance of such interventions in less severe heart failure is less clear [65, 70]. Updated guidelines for cardiac resynchronisation therapy advise confining its use to HFREF patients with ejection fraction below 35% [81].

**Fig. 2:**
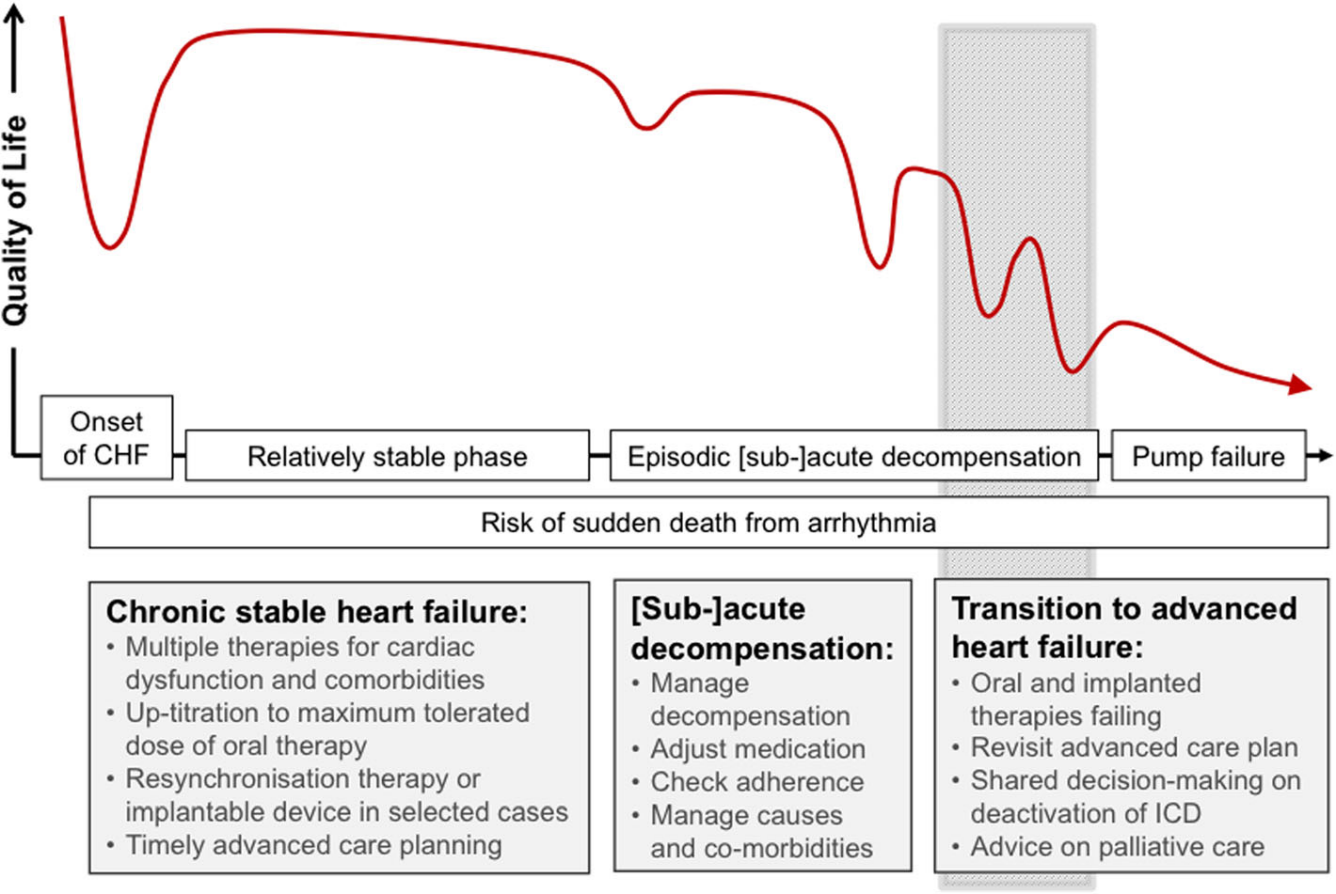
Clinical course of heart failure and its management. *Adapted with permission from McIlvennan* et al. [73]*.* ICD = implantable cardiac defibrillator

The clinical course of heart failure is characterised by progressive deterioration with bouts of (often unpredictable) acute or subacute decompensation from fluid overload, overdiuresis, non-adherence, infection or other intercurrent illness (Fig. 2). Sudden death may occur at any time due to arrhythmia, but more commonly there is gradual death from pump failure or, in a significant proportion, death from other cardiovascular or non-cardiovascular causes [65, 82, 83]. This disease trajectory makes heart failure an attractive test bed for those seeking a bioengineering solution for monitoring response to therapy and for predicting and pre-empting deterioration through close remote monitoring of biomarkers, thereby reducing expensive and disruptive hospital admissions. It is also potentially a way of obtaining accurate predictors of end-stage heart failure to inform timely conversations about end-of-life care. However, most people admitted to hospital *with* heart failure are not admitted *for* acute decompensated heart failure but for comorbidities and/or more general deterioration that are addressed tangentially or not at all by disease-specific guidelines and management protocols [52]. Because of misclassifications, hospital episode statistics tend to over-estimate the proportion of heart failure-related hospital admissions that could be prevented by treatment intensification in the outpatient setting [52].

Heart failure carries a five-year mortality of around 50% [84, 85]. Its prognosis is usually [52] but not invariably [84] reported to be improving; the disparity probably reflects a change in definition and case mix over time. Overall mortality figures mask considerable heterogeneity of outcome, which varies with setting at diagnosis (primary versus secondary care), first or subsequent hospital admission, diagnostic criteria (particularly whether and to what extent ejection fraction is reduced), co-morbidities (particularly diabetes and cognitive impairment) and degree of implementation of evidence-based therapies [80, 85, 86].

Importantly, people with heart failure, especially those with HFPEF, rarely die *of* heart failure, and the proportion of non-cardiovascular deaths in HFPEF is rising [82, 83]. In one large primary care cohort, only 26% of deaths in people with heart failure could be attributed to cardiovascular or cerebrovascular causes and 12% to heart failure, and there was no improvement in overall survival over time [82].

Heart failure management is delivered by multi-disciplinary teams which generally include cardiologists and hospital-based specialist nurses plus primary care clinicians (GP, pharmacist, community nurse) and (where necessary) palliative care specialists [87]. Communication and co-ordination between team members is critical. A large qualitative study, including over 200 interviews with staff in 50 centres across Canada, explored multi-disciplinary care of advanced heart failure and found that a high degree of adaptive behaviour (what Mol has described as “tinkering” [88]) was needed from the various clinicians to respond to patients’ emergent needs and achieve the best outcome in a particular set of circumstances [87].

### Heart failure as a lived experience

There is an extensive literature from qualitative and questionnaire studies on what it is like to live with heart failure [45, 89–97]. Because heart failure produces profound tiredness, shortness of breath, weight gain (from fluid retention) and unpredictable life-threatening acute crises, it typically has a life-changing impact on the individual’s psychological well-being, independence and ability to undertake activities of daily living. The economic and social burden of heart failure is high because of reduced ability to work and high demands on the time and commitment of partners and carers [92, 93, 98].

The initial diagnosis of heart failure engenders a sense of disruption and hopelessness from which patients recover variably [91, 94, 95, 98, 99]. In general, patients’ knowledge about heart failure is poor; many find the condition bewildering and explanations from professionals confusing or unforthcoming [91].

Chronic heart failure has three main impacts on daily life, which appear to be common across different ethnic and social groups. First, there is social isolation – arising from lack of energy to join in social events, the unpredictability of tiredness and physical weakness, and a feeling of abandonment by family and/or friends. Second, there is living with fear, including the fear of dying whilst asleep (common in patients with paroxysmal nocturnal dyspneoa). Third, there is loss of control, linked to unpredictable fluctuations in health, blood pressure, ease of breathing, sleep and the tyranny of complex medication regimens – and in the longer term to loss of independence, financial insecurity and loss of participation in decision-making [92, 98]. Social isolation may be worsened by medication regimens (e.g. diuretics producing urgency and frequency of urination).

There is limited evidence from small studies that women with heart failure may feel insecure and experience guilt and anxiety from *“being a burden”* [100] – but also that they may be more likely to come to terms with their heart failure than men, who may hold an unrealistic belief that the condition will improve or stop deteriorating [45, 93].

Some people cope by denying their heart failure and/or deliberately avoid learning about it [45, 92, 94]. One review classified patients on a continuum from *“strategic avoiders”* through *“selective deniers”* and *“well-intentioned self-managers”* to *“advanced self-managers”* [94]. Under-reporting of acute symptoms to health professionals is common [101]. Whilst patients often wish to obtain specialist help in an acute exacerbation, some may be unaware whom to contact, unable to navigate the system or unwilling to bother the doctor or nurse; in addition, fear of hospitals may deter people from seeking help [91].

Whilst hopelessness is common, adjustment to the diagnosis of heart failure occurs when and to the extent that patients are able to make sense of their illness experience, accept its (often poor) prognosis and enact practical strategies for living with the condition [45, 95, 99]. Coping strategies for living with heart failure include sharing experiences with others in a way that is socially and culturally congruent; gaining practical and emotional support from family and peers; and learning to adapt one’s expectations and lifestyle to changing circumstances (e.g. taking things more slowly, changing hobbies) [92, 95, 98].

The main influences on people’s ability to cope and care for themselves appear to be awareness and knowledge (including system knowledge about how to access and navigate services); absence of co-morbidity (especially depression); quality and continuity of professional care (including the clinician-patient relationship) [99]; quality of personal relationships (especially the extent to which partners are able to understand the condition, engage with its day-to-day management and ameliorate the person’s sense of being a burden); and quality of social support [92, 95, 97, 99].

Social support from family and lay networks can be thought of as having four interacting elements: emotional support, instrumental or practical help, provision of information and support with appraisal (that is, with evaluating one’s situation and the effect of one’s actions or inactions) [97].

The clinical course of heart failure makes conversations about end of life care important. Most heart failure patients would prefer such discussions to be physician-initiated and to commence early – certainly well before severe deterioration or clinical crises [102]. Because sudden death can occur at any time, some clinicians believe this risk should be discussed proactively [103]. Uncertainty of prognosis is often inherent, though palliative care scholars argue that such uncertainty should be embraced and woven into an evolving discussion in the context of a long-term therapeutic relationship [104].

### Self-management in heart failure

Self-management education programmes, whose aim is *“to enable patients to assume a primary role in managing their condition: monitor symptoms, adjust medications and determine when additional medical attention is necessary”*, have been shown to reduce hospital admissions and increase compliance with medication in heart failure [44] and (in more recent studies) to extend overall survival and improve quality of life [24]. Self-management in such programmes is taken to mean complying with prescribed medication; monitoring symptoms, blood pressure and weight; adhering to a low-sodium and fluid-restricted diet; stopping smoking; taking regular exercise and seeking timely advice from health professionals [24, 44].

However, not everyone with heart failure is able or willing to engage in these activities [91]. The physical manifestations of heart failure, which are typically *“vague, episodic and debilitating”* [45], have a significant effect on people’s ability to self-manage [105]. Balancing the demands of the illness and its treatment with the desire to live a normal life is a constant struggle, sometimes made worse by fragmented services and time-consuming hospital visits [106]. Depression, anxiety and cognitive impairment (including acute confusional states) are all much commoner in people with heart failure than in an age-matched population, and these conditions are associated with lower compliance with self-management programmes [18].

A recent meta-analysis of individual patient data from trials of self-management in heart failure included some telling subgroup analyses: self-management reduced hospital admission only in patients younger than 65 years (an important finding, since the mean age of people with heart failure is 75 [53]); in patients with moderate or severe depression it was associated with significantly *reduced* survival [24].

A realist review of heart failure self-management programmes, which addressed the question “what works for whom under what circumstances?” using mainly qualitative methods, identified six key mechanisms that have consistently been associated with programme success (in terms of improving self-management activities): mobilising high-quality, personalised support from health professionals; improving patient well-being; improving patient and carer understanding of heart failure; extending the network of people (lay and/or professional) involved in self-management and supporting people to use technologies [46].

Another realist review focused on the role of telehealth interventions in supporting self-management in chronic illness (including but not limited to heart failure) [47]. The authors identified three underlying mechanisms by which telehealth could support self-management: supporting *relationships* (with professionals and/or peers); successful *integration* of the technology into the patient’s everyday life; and making symptoms and biomarkers *visible* (thereby improving the patient’s knowledge, motivation and self-efficacy; mobilising support from the patient’s wider network; and reinforcing self-management behaviours). Another theoretical paper reviewed the various psychological theories (e.g. cognitive-behaviour theory, adult learning theory) on which the assumptions of telehealth-supported self-management are based [96].

### Telehealth technologies and services for heart failure

Telehealth means remote communication or monitoring between a patient and a clinical service using technologies. It is a heterogeneous term and implies technology (ies) plus a service in which they are embedded [7]. *Remote communication* supports conversations (either open-ended or structured, via telephone or video), and potentially offers scope for developing and maintaining therapeutic relationships. *Remote monitoring* relies on the principle that systematic monitoring of predefined variables will enable early detection of clinical deterioration and timely intervention. Four types of data may be monitored:

- Symptoms (e.g. breathlessness, fatigue);
- Behaviour or events (e.g. compliance with medication and fluid restriction, physical activity, falls);
- Biological data captured non-invasively via external devices (e.g. body weight, body impedance, blood pressure, heart rate, ECG, blood oxygen, blood glucose, natriuretic peptides);
- Biological data captured invasively via implantable devices (e.g. intracardiac or pulmonary artery pressure, dysrhythmias).

Technologies for collecting the above data are many and varied. They include sensing devices (typically blood pressure monitors, weighing scales, pulse oximeters), simple telephones (with human-human interaction); telephone-based voice-response systems (human-machine interaction); video consultation (with or without transmission of vital signs); interactive portals on laptops or smartphones; wearable sensors and implantable sensors [21, 38, 64]. All imply the presence, at some stage in the pathway, of humans on the receiving end of signals and a service infrastructure in which those humans can respond to a problem and/or refer it on. Links with the electronic patient record may be automated or require manual data entry.

Anker et al. have suggested a broad taxonomy of telemonitoring devices based on two things: whether data are sent synchronously or asynchronously and whether professional clinical input occurs immediately or only during office hours [64]. They point out that remote monitoring raises medicolegal questions (if data are collected but not immediately analysed, what degree of delay is acceptable?); who takes responsibility for failures in transmission of data?; to what extent is a ‘exception’ approach – in which clinicians are only alerted if variables go outside pre-set parameters – appropriate and defensible?; and financial concerns (who pays for which aspects of the service, and how?), to which answers are currently unclear [64].

Some telehealth interventions feed back directly to the patient with educational messages (e.g. prompts to exercise) and/or decision support for self-management (e.g. suggesting a change in drug dosage). Such interventions are depicted as potentially more efficient (since drug dosage can be changed promptly by an “activated” patient or carer) and more cost-effective (since clinician input is reduced), but they also place high demands on the patient or carer [7].

Conventional telehealth using non-invasive monitoring technologies is being supplemented or superseded by the use of implantable devices, which raise additional clinical, technical, operational and ethical challenges [4, 5, 11, 21, 23, 36, 38, 65, 68]. In some cases, a device is specifically implanted to measure a particular biomarker (e.g. pulmonary artery pressure); in others, the device is implanted for other reasons (e.g. an implantable defibrillator), allowing biomarkers to be measured in parallel. Notwithstanding the future potential of such devices in some circumstances, hospital admission in people with heart failure is often triggered by co-morbidities and psychosocial factors; telehealth devices and services that fail to address these influences will have limited impact [64].

The sociological literature on telehealth emphasises that the devices (like all technologies) are not neutral. Rather, both standards (what gets measured and how frequently) and values (assumptions about what is good – for example, that heart failure patients should take responsibility for self-management) are built into them. Whilst some technologies can be reprogrammed and/or used flexibly at the discretion of the clinician, non-use of telehealth devices can sometimes be explained in terms of mismatches between the inbuilt values and standards of devices and those actually held by patients and staff. The testing of telehealth technologies in randomised trials (for which strict protocols are usually required) may fail to capture the judicious and flexible use of such technologies in a real-world clinical setting.

### Trials of telehealth in heart failure

Over the past 20 years, randomised trials of telehealth in heart failure have appeared at a rate of several per year; a new meta-analysis attempting to synthesise such trials is published every few months [14–17, 19, 21–23, 25–32, 34–39, 107, 108]. This vast, complex and statistically daunting literature has been summarised and critiqued in detail elsewhere [5–7, 21, 33, 38, 39, 55, 64, 65, 68, 70]. In this section, we summarise key points from published reviews and consider the question of why so many trials and secondary analyses have produced so few clear recommendations for practice and policy.

The single most striking feature of experimental trials of telehealth in heart failure is their heterogeneity. As the above-referenced reviews have noted, different trials included widely differing patient samples in terms of demographic variables (especially age cut-offs), severity and stability of disease (especially the cut-off value for HFREF and whether patients with HFPEF are included), exclusion criteria (especially co-morbidities), and recruitment route (outpatient clinic, post-acute hospital admission, primary care); they collected different kinds of data using different devices combined with different packages of human support in different organisational settings; they compared these interventions with different control arms (especially, different definitions of ‘usual care’); and they applied different primary and secondary outcome measures (total or heart failure-specific hospital admission, all-cause or cardiac-specific mortality, and quality of life).

Unsurprisingly in the circumstances, different trials of telehealth in heart failure got different results. Meta-analyses of such trials, claim critics, have tended to take too little account of inter-study differences and give too much weight to flawed primary studies [6, 33, 38, 39, 64, 65]. Whilst there is much residual confusion, five consistent messages emerge from the experimental literature:

First, published trials of telehealth in heart failure appear to be positively skewed by publication bias [33]. In one review, 60 studies reported positive outcomes (almost all of them only weakly positive) and only one a (weakly) negative outcome [33] – a statistically implausible result. Despite the beneficial effects of telehealth reported in several meta-analyses [16, 19, 22, 23, 25–28, 30, 35, 38, 107, 108], a number of large, recent randomised controlled trials have demonstrated no statistically significant impact from comparable telehealth interventions and patient groups [9, 10, 12, 13] (though one recently published large trial showed positive benefit [11]). A Cochrane review of “interactive” telehealth (involving direct contact between a patient and a clinician) published in 2015, in which methodologically weaker studies were excluded using a risk of bias tool, showed no overall benefit over usual care on all-cause mortality or hospitalisation in the heart failure sub-sample [17].

Second, recruitment of participants to trials of telehealth for heart failure is usually poor and sometimes abysmal. In one trial of telehealth that included but was not limited to heart failure, 80% of approached patients refused to participate; the most common reason was preferring a home visit from a nurse [56] – a finding that resonates with a more recent, larger trial in which one reason for non-participation was fear that a highly-valued existing service might be withdrawn [58]. In another trial, 65% of patients were deemed ineligible (most commonly because face to face home visits were considered clinically necessary) and of the rest, 75% declined to participate [57]. In a review of heart failure telehealth studies that reported recruitment data, on average, two-thirds of eligible participants agreed to try telehealth and of those, a fifth withdrew during trials (most commonly because they were unable to use the device or did not wish to) [32]. The lower the recruitment success in trials of telehealth, the more questionable the external validity of the findings.

Third, the impact of telehealth on reducing mortality, preventing avoidable hospital admission and improving quality of life appears to be highest in high-risk patients recruited after acute admission for heart failure (around one-sixth of the total heart failure population) [19, 21], and attenuates with length of follow-up [19]. This makes sense clinically, since stable heart failure is less likely to require frequent adjustment of medication. It follows that telehealth support is unlikely to improve outcome in the majority of people with heart failure and that careful case selection will be key to achieving clinical and cost-effectiveness.

Fourth, the efficacy of remote telehealth monitoring appears to improve with the number of variables monitored, the frequency of monitoring and the timeliness of human remote support [19, 22]. Inclusion of symptom questions (e.g. subjective breathlessness) and monitoring of pulse rate and rhythm both significantly enhanced the benefit of remote telehealth monitoring [19]. In one meta-regression analysis, the single largest contributor to the success of telehealth interventions was active adjustment of medication within one day of a key change in vital signs [22]. Whilst invasive monitoring of intra-cardiac biomarkers is *theoretically* more accurate than non-invasive monitoring, trials of these newer forms of telehealth have produced inconsistent findings to date (though several studies are ongoing) [7, 11, 23, 65].

Fifth, telehealth shows greater benefit when “usual care” is sub-optimal [7, 109]. The TIM-HF trial, for example, randomised 710 patients with exceptionally well managed chronic stable heart failure to high-quality expert remote support or high-quality usual care and found no significant differences in outcome between the groups [10]. Some have suggested that telehealth might come into its own when care provided to the control arm falls below the gold standard used in TIM-HF [109]. A consistent finding is that when trials showed a benefit of telehealth compared to usual care, participants in the telehealth arm were found to be on higher doses of disease-modifying agents than those in the control arm [7].

### Cost

Pandor et al., who undertook one of the most comprehensive and rigorous systematic reviews on the remote management of heart failure, also included a review of economic evaluations [21]. Using studies published up to early 2012, they produced a Markov model to assess the cost-effectiveness of remote monitoring and structured telephone support, based on both randomised trials and observational cohort studies. In this model, they found remote monitoring during office hours to be cost-effective (incremental cost-effectiveness ratio [ICER] £11,873 per quality-adjusted life year [QALY]). Structured telephone support with human contact was not cost-effective (ICER £228,035 per QALY) and when structured telephone support using a machine interface was compared with usual care, the latter emerged as dominant (i.e. more cost-effective). The authors considered that they had insufficient data to evaluate the cost-effectiveness of 24/7 remote monitoring or monitoring using implantable devices.

A number of systematic reviews of economic evaluations of telehealth in heart failure have been published since Pandor et al.’s review. One considered the primary economic data to be too poor to support any firm conclusions [40]. Another considered the impact of patient adherence on costs of telehealth and also failed to demonstrate a consistent relationship between these variables [41]. A third found wide variation in cost-effectiveness across studies, with simpler technologies (e.g. monitoring only one vital sign) proving more cost-effective than complex ones – but also found that more recent studies tended to show better cost-effectiveness (because equipment is getting cheaper) [42]. Liu et al. produced a new Markov model showing that telehealth was most cost-effective (indeed, cost-saving) in the most high-risk patients, though all estimates were highly sensitive to the costs of hospital admission and length of stay [43]. In short, cost estimates vary widely because of heterogeneity of samples and the numerous interacting variables that play out differently in different settings.

### Implementation studies of telehealth

By implementation studies, we include preliminary work to optimise technologies and service routines before undertaking randomised trials; process evaluations of the intervention arms of such trials; and real-world studies of the practicalities and challenges of introducing and sustaining telehealth services. Many such studies are largely atheoretical and written up as lists of “barriers” and “facilitators” [32, 50, 58, 61, 62], though some depict a complex system with multiple interacting influences including people, technologies and both personal and professional routines [7, 47, 89, 110, 111].

Table 2 lists the many factors to which poor uptake of telehealth in heart failure has been attributed. Despite frequent acknowledgement of these factors in the literature, empirical research into telehealth has tended to be narrowly focused on describing and trialing particular technologies in experimental designs without systematically studying the personal, professional, organisational, financial and regulatory context affecting their acceptance and routinisation (a bias that has been exacerbated rather than resolved by the increasing use of cloud computing and big data) [5]. To our knowledge, no implementation study of telehealth in heart failure has described and tested a specific theory of change.

**Table 2:** Factors shown to account for poor uptake of telehealth in heart failure

PATIENT FACTORS	Low motivation – perhaps due to belief that the technology will have no benefit over existing approaches to care (“relative advantage”)
	Preference for a face to face encounter
	Inability to use the technology (including limitations of health impairments)
	Inability or unwillingness to take action in response to data or remote instructions
	Lack of confidence in own ability to use the technology or the service (self-efficacy)
	Fear that engaging with telehealth will lead to exclusion from a valued traditional service
STAFF FACTORS	Absence of champions
	Dislike of new clinical routines (including increased workload)
	Dislike of new clinical interaction (i.e. prefers face-to-face encounters)
	Belief that relationships and therapeutic interactions will be compromised
	Perception that their clinical expertise is being marginalised
	Perception that there is no value for them in the new way of working
	Inability to use the technology (including inability to remember password)
TECHNICAL FACTORS	Technology unreliable (including too slow, or interrupted)
	Technology too difficult to use
	Technology doesn’t fit / gets in the way in patient’s home
	Technology (and/or the routines for using it) too inflexible
	Inadequate IT infrastructure including absence of high bandwidth connectivity
	Inter-operability problems (especially with electronic patient record)
	Inadequate helpdesk or technician support
TEAM/SERVICE FACTORS	Lack of clarity about who will interpret and act on remote monitoring data
	Poor integration of the telehealth support role with wider team and service roles
	Poor working relationships between providers
	Insufficient staff
	Absent, inadequate or delayed staff training
	Lack of guidance on which patients/conditions are suitable for telehealth consultations
	Lack of a clear and integrated referral pathway
	Lack of (or inadequate) participation of staff in the implementation process
	Lack of timely feedback on the success of the service
	Programme dependent on a single individual with inadequate succession planning
GOVERNANCE AND REGULATORY FACTORS	Concerns about data protection and privacy
	Inadequate supporting policy and legislation
	Opposition (or lack of active support) from professional bodies or defence societies
FINANCIAL/BUSINESS FACTORS	Lack of a plausible business case
	Lack of clear strategy
	Unrealistic financial reimbursement
	Unsupportive policy context

*Compiled from various sources* [5, 7, 48, 60, 61, 63, 110, 111, 113, 122, 123]

Some research teams have included a preliminary co-design phase in which front-line clinicians and service managers (and sometimes patients and carers as well) work to design and/or refine the technology and shape the service routines in which it will be used. Triantafyllides et al., for example, describe such a process in relation to the SUPPORT-HF randomised trial, which involved patients entering structured symptom summaries and physiological data onto a tablet and (in the intervention arm) receiving remote advice and education [112]. The co-design phase with patients led to several upgrades (such as enhanced ‘helpdesk’ support, remote activation and de-activation of features of the application, and regular data synchronisation to address intermittent internet connection failure).

Wade et al. interviewed 36 clinicians involved in different telehealth projects across Australia [111]. Whilst the authors initially anticipated organisational and regulatory issues to be important factors in accounting for low uptake of telehealth (as indicated in Table 2), their data supported a single key factor – clinician acceptance – that could make or break such a service, given adequate technology, resources and demand. In their analysis, acceptance depended on the belief that telehealth was effective, safe and “normal” – and such beliefs were in turn strongly influenced by local champions [111]. In this study, champions worked by enthusiastically *promoting* telehealth, *legitimating* telehealth and *building relationships* [113]. Other studies have also identified clinician non-acceptance as the most important single explanation for low uptake of telehealth [51, 60, 101, 110]. A Dutch qualitative study found that some clinicians used their physical senses (sight, touch, smell) and “gut feelings” in assessing very ill patients, and were concerned that remote monitoring (and even video consultations) would lead to loss of this rich data [101].

Action research employing the principles of socio-technical co-design allows iterative development of a technology and the service to which it is linked, including negotiating the underpinning values and standards that will be built into the technology, as well as engaging staff (and, sometimes, patients) in shaping the pathways and routines in the technology-supported service. Whilst such an approach is theoretically appealing, the research literature evaluating its use in heart failure services is sparse.

We found only one relevant action research study, from the UK, which included patients with chronic obstructive pulmonary disease as well as heart failure [114]. An initial interview phase fed into a series of action research cycles in four sites, all of whom identified the first priority as establishing clear referral criteria and clarifying the telehealth pathway (including how to deal with patients who had become dependent on telecare but are no longer deemed to need it clinically). Other priority actions addressed in the action research were improving patient assessment and review, improving monitoring and triage, improving data sharing and access, raising awareness of telehealth, improving evaluation and securing further funding. In this study, “early wins” and a sense of partnership appeared critical to overcoming clinician resistance and maintaining buy-in [60].

## Discussion

### Summary of findings

Heart failure is a chronic disease, mostly affecting the elderly and managed in the community. It is characterised by periodic acute exacerbations for which (potentially avoidable) hospital admission is common and by progressive deterioration. Modern management of heart failure with reduced ejection fraction (HFREF) involves complex drug regimens, including up-titration of multiple medications to achieve the maximum tolerated dose, and/or technologically advanced devices, as well as active involvement of patient and carers. Heart failure with preserved ejection fraction (HFPEF) is increasingly common, less amenable to up-titration of medication and less widely researched. Modifiable risk factors and co-morbidities (hypertension, coronary heart disease, valvular heart disease, diabetes, obesity, smoking, history of myocardial infarction, cardiotoxic chemotherapy) present both opportunities and challenges for telehealth interventions.

Heart failure is experienced by the patient as a serious, debilitating and unpredictable condition that engenders bewilderment, fear and hopelessness. It interferes in a major way with activities of daily living, imposes a high treatment burden and carries a real risk of death. The qualitative literature underscores the importance of family and social networks in supporting individuals to live with heart failure. Self-management appears to improve functional status, quality of life and survival in trial populations but there is now strong evidence that the oldest and sickest patients appear not to benefit from it, have limited capacity to undertake it and may even be harmed by it.

Telehealth is an umbrella term that embraces multiple technologies, used variably by different services, to communicate with and monitor heart failure patients. These technologies are developing rapidly and becoming more invasive. They are part of wider networks of people and technologies; they generate data that must be processed, interpreted and acted upon by humans – and, in current iterations, they address comorbidities only to a very limited degree. They embody standards and values which may not be immediately evident but which could potentially conflict with those held by patients and professionals.

Several high-quality randomised trials have demonstrated the superiority of telehealth over usual care in patients with severe and unstable heart failure – though even in this select sub-group, telehealth’s edge is evident only when intensively delivered and when “usual care” is suboptimal. The literature is muddied by poor-quality, underpowered trials with weakly positive results, synthesised (sometimes) in misleading meta-analyses that overlook publication bias and lump different denominator populations, devices and service models together inappropriately. Low recruitment to telehealth trials raises questions about the external validity of results. Implantable monitors are a rapidly developing technology on which the evidence base is still accumulating.

The somewhat sparse economic literature resonates with findings from high-quality clinical trials: that telehealth interventions are most cost-effective where they are most effective – in unstable patients who are at high risk of hospital admission. They also raise the intriguing question of whether simpler monitoring schedules, even though less *clinically* effective, may be more *cost*-effective.

The literature on implementation of telehealth in heart failure can be summarised in a long and perhaps unsurprising list of barriers to (and conversely, facilitators of) success, of which clinician resistance appears to be particularly significant (Table 2). Co-design with front-line users can help optimise technologies and service routines prior to randomised trials. Action research with a strong co-design component appears to offer promise for real-world implementation but there are, as yet, few such studies in the literature.

### Synthesis

We have surfaced a number of tensions in the literature, which we believe are the key to explaining variation in uptake and sustainability of telehealth services.

There is a tension between heart failure as an isolated textbook condition, amenable to standardised management by evidence-based guidelines, and the much commoner phenomenon of heart failure coexisting with multiple co-morbidities that impact on the patient experience and key outcome measures (hospitalisation, mortality). The bioengineers’ vision that continuous biomedical data will allow pre-empting of deterioration (and hence, hospital admissions) and systematic improvements in prognosis may materialise for some patients but for others it will remain unrealised as risk factors and comorbidities increase noise in the system.

Another tension is between an intensive telehealth monitoring service that collects data on multiple symptoms and biomarkers (more clinically effective) and a more basic service that focuses on one or two easily-measurable parameters (more cost-effective). The decision to provide enhanced versus basic telehealth support could potentially be made clinically on a case by case basis, thereby optimising the trade-off between clinical and cost-effectiveness of the service, though we did not identify any published research that had tested such a hypothesis.

There is also a tension between the activated, empowered, self-managing patient assumed in many hypothetical models of telehealth and the tired, hopeless, frightened and bewildered individual identified in qualitative studies of the patient experience. Also of note is the chronically anxious or depressed, and/or acutely confused, patient who features strongly in the denominator population of many heart failure studies. Policymakers who view telehealth-prompted self-management uncritically as the technological solution to the rising prevalence of heart failure in an ageing population should note the evidence reviewed above that this is at best a partial solution for younger and fitter sub-populations.

A fourth tension is between heart failure management as a “cold” biomedical practice *“dominated by technical, objectifying and causal reasoning about the body and its diseases”* (page 25 [101]), focused on data exchange and oriented to improving efficiency and quantitative outcome measures (mortality, length of stay and so on) – and as a “warm”, relationship-based, adaptive practice that engages with the patient’s unique predicament, acknowledges his or her vulnerability and emotions, and seeks to provide continuity of personal care. The two approaches are not, of course, mutually exclusive, and a successful heart failure service may conceivably combine both, adapting the care package judiciously to each patient’s needs and preferences. The tension between “cold” and “warm” telehealth highlights that seemingly technical and economic choices (about which devices to purchase for a heart failure service) mask *value-based* choices about what kind of care patients deserve.

Finally, there is the tension between fixed and agile programmes. The preferred research approach of the randomised controlled trial, in which both the technology and the activities it supports are more or less fixed (though, as noted above, a trial may be preceded by an iterative developmental phase), means there has been limited opportunity to study the process by which technical and clinical staff, as well as patients and carers, “tinker” with the technology to produce a customised package that is fit for purpose for a unique set of circumstances, and to modify that package as the patient’s condition fluctuates.

### Comparison with existing literature

Ours is not the first literature review of telehealth in heart failure that sought to make sense of a complex field and explore its many interactions and nuances. Conway et al., Dierckx et al. and Gurné et al. before us (and to some extent, other authors too) have resisted the temptation to combine findings from heterogeneous trials of telehealth into a dubious single ‘effect size’ and instead engaged critically with shifting definitions of the condition and multiple sources of heterogeneity (pathophysiology, co-morbidity, case mix, devices, services, outcome measures) and with the diverse policy contexts in which decisions about telehealth are made locally and nationally [6, 7, 36].

We believe we have added to their work by using a formal, hermeneutic methodology and incorporating a wide range of literature beyond randomised trials. Drawing on Wittgenstein, Boell and Cecez-Kecmanovic distinguish between puzzles or problems that require *data* and those that require *clarification and insight* [67]. Telehealth in heart failure is a “paradigm case” not only practically (it illustrates the multiple complex challenges facing any telehealth programme) but also methodologically (because of its complexity, the field will not be illuminated – and may sometimes even be made murkier – through data-focused review methods such as charting, categorising and meta-analysis).

The tensions listed above both illustrate and underscore the ARCHIE standards developed previously by our own team: telehealth and telecare services must be *Anchored* in a detailed understanding of what matters to the patient; *Realistic* about the natural history of their condition; *Co-creative* so as to evolve and adapt solutions with users; *Human*, supported through interpersonal relationships and social networks; *Integrated*, through attention to mutual awareness and knowledge sharing; and *Evaluated* to drive system learning [115].

### Unanswered questions

The extensive empirical literature and the somewhat sparse theoretical literature summarised above leave a number of research questions unanswered. Drawing partly on a paper by Pols [101], we identify five that are particularly relevant to the tensions that emerged from our own hermeneutic review.

First, what is excellence in the clinical management of heart failure? If technologies embody values, it is surely necessary to surface and debate our professional values about good care which – often implicitly rather than explicitly – inform and drive the design of telehealth technologies. How important is relationship-based care, for example, for heart failure patients in general and, more specifically, for particular patients or patient groups? How should we manage the trade-off between evidence-based guidelines (which relate to sub-populations and can be built into algorithms) and personalisation of care (which accommodates the unique features of the individual and cannot)?

Second, what kinds of knowledge are needed to deliver excellent care (and how do clinicians’ knowledge needs change with circumstances)? Different telehealth technologies bring different kinds of knowledge into play. A device that transmits accurate physiological data (but nothing else) informs a very different kind of clinical decision-making from a technology that transmits a view of the patient’s face and the family living room along with the sound of stories, laughter and arguments between spouses (but few objective data). Because new forms of telehealth surface new forms of knowledge, they have profound implications for what care *is* and how services must be organised to deliver it.

Third, what is the nature of “complexity” in heart failure and its management, and what are the implications of this for the design of technology-supported services? This paper has begun to tease out the different ways in which the management of heart failure (especially but not exclusively when done remotely) is clinically, logistically and technically complex. Further research could explore this complexity in more depth, perhaps starting with the group most neglected in studies of heart failure to date – the oldest, most frail patients with multiple comorbidities and additional vulnerabilities.

Fourth, in the light of the finding from a large, well-conducted qualitative study that almost all patients in heart failure clinics need considerable customisation of care [87], what is the nature of this “tinkering” and what are the implications for those who (with the best of intentions) seek to make heart failure care more automated, more standardised, more guideline-driven and more efficient? The use of telehealth does not preclude tinkering, nor does it imply unquestioning adherence to guidelines. But the judicious, patient-by-patient customisation of the telehealth package to support compassionate, individualised evidence-based care appears to have received little research attention to date. If such tinkering were not merely permitted but encouraged and resourced, would this overcome resistance of some front-line staff to telehealth in heart failure?

Fifth, for what aspects of care has the potential of telehealth not yet been (fully) explored? For example, what kinds of comorbidities might be managed remotely, and how? How and in what circumstances might a primary focus on comorbidities improve the heart failure that is secondary to them? Could remote consultations be used to help provide education, review compliance and rationalise medication in selected heart failure patients? Could some aspects of advance care planning be done through this medium?

Finally, how could we use co-design and action research methodologies more effectively in the development and implementation of telehealth services? There is much evidence that introducing technologies in an organisation is a social process that depends on values, mindsets and engagement [116]. It is also an evolutionary process (sociotechnical systems are grown, not built), hence best achieved by early and active input of front-line workers into the [re] design of work routines. Similarly, if we expect patients to accept and engage with telehealth services, we should involve them in the design of these services. It is time to extend the limited evidence base on how this might best be undertaken.

## Conclusion

This hermeneutic review has highlighted the clinical complexity of heart failure and its management; its profound physical and psycho-social impacts and the significant treatment burden placed on the patient and family; the benefits and (in older and sicker patients) potential drawbacks of self-management; the wide range of rapidly-evolving technologies and services that come under the umbrella of telehealth; the vast number of experimental trials of telehealth whose findings are ambiguous, interpretations contested and costs highly variable; the many challenges of implementing telehealth in real-world settings; and the widespread (but by no means universal) resistance to telehealth among patients and clinicians.

We have synthesised our findings into a number of key tensions – between tidy, ‘textbook’ heart failure (seen more often in secondary care) and its messier, comorbid reality (which predominates in primary care); between basic and intensive telehealth; between patients who are activated, empowered and linked to an extensive network of lay carers and those who are bewildered, downtrodden and socially isolated; between “cold” and “warm” telecare; and between fixed and agile programmes of care.

We believe that, despite a number of ongoing, well-designed randomised trials of new technologies and care models, the research landscape in this field has become somewhat stagnant. Our suggestions for new topics of research deliberately seek to address different questions using different methodologies. For primary research, we encourage greater use of theory-informed qualitative methods, especially when considering the implementation of telehealth in organisations, and of co-design methods, action research and agile development of technologies and programmes. For secondary research, we believe hermeneutic review should be used much more widely to illuminate and clarify complex topic areas.

## Additional file

Additional file 1: Pathophysiology and classification of heart failure. Supplementary literature review. (DOCX 19 kb)

## Abbreviations

HFmrEF: Heart failure with mid-range ejection fraction
HFREF: Heart failure with reduced ejection fraction
HRPEF: Heart failure with preserved ejection fraction
ICD: Implantable cardiac defibrillator
ICER: Incremental cost-effectiveness ratio
NICE: National Institute for Health and Clinical Excellence
QALY: Quality-adjusted life year
