# Supplementary material for: Understanding heart failure; explaining telehealth – a hermeneutic systematic review
Source: BMC Cardiovasc Disord. 2017 Jun 14;17:156. doi: 10.1186/s12872-017-0594-2 (PMC5471857; doi:10.1186/s12872-017-0594-2)
Supplement: Additional file 1: — Pathophysiology and classification of heart failure. Supplementary literature review. (DOCX 19 kb) [file 12872_2017_594_MOESM1_ESM.docx]

# Additional file 1: Pathophysiology and classification of heart failure

Heart failure is not a single disease but a clinical syndrome whose pathophysiology varies with underlying cause, over time and in response to treatment. At some point in the natural history, ventricular filling or ejection is impaired. This causes exercise limitation manifested as breathlessness or fatigue, often accompanied by orthopnoea, paroxysmal nocturnal dyspnoea and ankle swelling. These symptoms (what the patients feels) are accompanied by signs (what the clinician observes). Classical signs include a raised jugular venous pressure, displaced apical beat and third heart sound. Heart failure symptoms and signs are harder to discern and interpret in the obese, the elderly and those with chronic lung disease, and they can resolve on treatment. For a firm diagnosis, there must be objective evidence of heart damage on investigations (echocardiography, MRI or isotope uptake studies) [1].

Acute heart failure is characterised by inability of cardiac output to meet the immediate needs of metabolising tissues. In chronic heart failure, particularly when treatment is optimised, cardiac output can be normal and even increase with exercise, so that limitation is only experienced on peak demand. Exercise limitation underpins the New York Heart Association functional classification of symptom severity; namely Class I (no limitation of physical activity); II (slight limitation); III (marked limitation) and IV (unable to do any activity without discomfort). [2]

The evolutionary model of heart failure describes how myocardial damage – e.g. as a result of infarction or cardiomyopathy – reduces the proportion of blood pumped out with each contraction of the ventricle (“heart failure with reduced ejection fraction”, HFREF, commonly termed left ventricular systolic dysfunction). The resulting lowering of cardiac output triggers a reflex neurohumoral response (which originally evolved to restore homeostasis in situations of blood loss, dehydration or sepsis), thereby activating the renin-angiotensin system (RAS). This increases vascular resistance and fluid retention, which (in the context of a damaged heart) is maladaptive, putting further strain on the failing heart. Many therapies for HFREF work by counteracting RAS activation, but they have the unintended consequence of rendering people vulnerable to those same primitive stressors of blood loss, dehydration or sepsis, so reducing patients’ resilience in the face of what would otherwise be minor intercurrent illness.

In some patients, the primary insult is the insidious effects of hypertension, obesity or diabetes. Metabolic damage from these conditions leads to thickening, stiffening and fibrosis of the ventricle, impaired relaxation during the cardiac cycle and an increase in filling pressure – which in turn leads to pulmonary and peripheral oedema (“heart failure with preserved ejection fraction”, HFPEF, formerly known as diastolic heart failure). Patients with HFPEF comprise at least half of all heart failure cases and have a similar mortality [3, 4]. These individuals have been relatively neglected in intervention studies but account for a high and rising proportion of clinical workload, especially in primary care. Therapies that improve prognosis in HFREF tend to be ineffective in HFPEF, and acute hospital admission improves survival in the former but not the latter [1, 4].

The arbitrary cut-off value defining a ‘reduced’ or ‘preserved’ ejection fraction has varied historically, being 40% in some studies and 45% in others. Latest guidelines from the European Society of Cardiology have dealt with this by defining an additional category, Heart Failure with mid-range ejection fraction or HFmrEF, classifying individuals with an ejection fraction of 40-49% in a ‘grey area’ for prognosis and management [5]. The new categorisation is intended to inform more precise research studies but to our knowledge has not been used to classify participants in telehealth trials published to date.

1. McDonagh TA, Gardner RS, Clark AL, Dargie HJ: **Oxford Textbook of Heart Failure**. Oxford: Oxford University Press; 2011.

2. American Heart Association website: **Heart failure classification.** . Accessed 6.2.17 on [http://www.heart.org/HEARTORG/Conditions/HeartFailure/AboutHeartFailure/Classes-of-Heart-Failure_UCM_306328_Article.jsp - .WErA6XecZsY](http://www.heart.org/HEARTORG/Conditions/HeartFailure/AboutHeartFailure/Classes-of-Heart-Failure_UCM_306328_Article.jsp#.WErA6XecZsY) 2017.

3. Hogg K, Swedberg K, McMurray J: **Heart failure with preserved left ventricular systolic function; epidemiology, clinical characteristics, and prognosis**. *J Am Coll Cardiol* 2004, **43**(3):317-327.

4. Owan TE, Hodge DO, Herges RM, Jacobsen SJ, Roger VL, Redfield MM: **Trends in prevalence and outcome of heart failure with preserved ejection fraction**. *New England Journal of Medicine* 2006, **355**(3):251-259.

5. Ponikowski P, Voors A, Anker S: **The Task Force for the Diagnosis and Treatment of Acute and Chronic Heart Failure of the European Society of Cardiology (ESC); developed with the special contribution of the Heart Failure Association (HFA) of the ESC. 2016 ESC Guidelines for the diagnosis and treatment of acute and chronic heart failure**. *Eur Heart J* 2016, **37**(27):2129-2200.
